# Transparency in Conflict of Interest Meta‐Research: Author Identification and Implications for Clinical Evidence

**DOI:** 10.1111/jep.70448

**Published:** 2026-04-23

**Authors:** Clovis Mariano Faggion

**Affiliations:** ^1^ Department of Periodontology and Operative Dentistry University Hospital Münster Münster Germany

**Keywords:** clinical decision‐making, conflict of interest, ethics, meta‐analysis, publishing/standards, research integrity, transparency

## 1 Introduction

Transparency is a cornerstone of research integrity and underpins trust in scientific evidence [1]. Beyond complete reporting of methods and results, transparency requires adequate disclosure of interests that may influence the design, conduct, interpretation or communication of research. Journals increasingly promote open science practices such as protocol registration, open data and preprint posting. Yet, empirical meta‐research indicates that disclosure of interests remains incomplete, inconsistent or difficult to interpret across biomedical research [2, 3].

Because systematic reviews and clinical practice guidelines directly shape clinical judgement and patient care, limitations in how conflicts of interest (COI) are assessed, disclosed, or verified have implications that extend beyond methodological rigour and into clinical decision‐making. Inadequate transparency about interests may therefore affect not only the interpretation of individual studies but also the evaluation of bodies of evidence that inform practice.

Meta‐research on COI typically aims to identify systemic patterns in reporting practices and associations between interests and research outcomes, rather than to assess individual misconduct. A recurring methodological question in this field is whether investigators included in such studies should be publicly identified or anonymized. This commentary examines when author identification may be methodologically relevant in COI meta‐research, the ethical and practical challenges involved, and how cautious, non‐accusatory reporting can support accountability without conflating transparency with blame.

## 2 Conflicts of Interest and Disclosure: Clarifying Terms

Discussions of COI often conflate distinct concepts. Disclosure of interests refers to authors' reporting of relationships or activities that may be relevant to a study; not all disclosed interests constitute conflicts. COI arise when secondary interests risk unduly influencing professional judgement [4]. Interests may relate to study funding or to individual authors and may be financial (e.g. consulting fees, industry funding) or non‐financial (e.g. personal relationships, professional affiliations). Lack of terminological clarity complicates both primary research reporting and meta‐research assessing such reports.

For example, an author reporting prior consultancy with a pharmaceutical company does not automatically indicate bias in their current work. It is a disclosure that allows readers to contextualise findings. Similarly, distinctions between individual‐level and study‐level interests are critical when assessing how funding or relationships may correlate with reported outcomes. Recognising these nuances enhances both methodological rigour and interpretability in meta‐research results.

## 3 Meta‐Research on COI and the Role of Author Identification

Most meta‐research on COI can be broadly categorised as prevalence studies (e.g. estimating how often interests or funding sources are disclosed) or association studies (e.g. examining associations between industry funding and favourable conclusions) [5, 6, 7]. Researchers often rely on publicly available sources such as journal articles, disclosure forms, trial registries, or payment databases.

Author identification becomes methodologically relevant when readers need to verify extracted information, contextualise findings, or understand recurring patterns across publications. For instance, anonymizing investigators as ‘Author A’ or labelling studies numerically prevents independent verification of whether disclosed interests align with information in public registries or across multiple publications. Identification can enhance interpretability and verifiability by allowing readers to assess the consistency and completeness of disclosures over time and across sources.

At the same time, author identification is not always necessary. Aggregate analyses addressing overall prevalence or broad associations may be interpretable without naming individuals, particularly when verification at the individual level is not central to the research question [8, 9, 10]. For example, a study estimating that 30% of systematic reviews report industry funding can yield meaningful insights without identifying each author. Decisions to identify authors should therefore be guided by study aims rather than a general presumption for or against anonymization.

## 4 Ethical and Practical Challenges

Concerns about reputational harm and legal risk contribute to editorial reluctance to publish studies that identify individuals, even when data are drawn from publicly available and independently verifiable sources [11]. These concerns are legitimate, particularly where errors or misinterpretation could unfairly damage professional standing. Consequently, many COI studies report findings in aggregate form, sacrificing granularity to mitigate potential harm.

Practical constraints also matter. Meta‐research studies may include hundreds of publications and thousands of authors, and disclosures are often reported inconsistently or only by initials. Fully linking individual authors to specific interests may require substantial resources and specialised data handling. Such efforts are often feasible only for targeted studies rather than large‐scale prevalence assessments. Acknowledging these constraints is important when formulating recommendations for reporting practices.

One possible middle ground is to offer investigated researchers the opportunity to verify factual information derived from public sources prior to publication, without granting veto power over inclusion. While this approach may reduce errors, selective non‐response is a risk. Verification should not be mandatory when the research objective is to assess systemic patterns rather than individual conduct. This strategy reinforces the principle that accountability in research is about improving systems, not assigning blame to individuals.

## 5 Ambiguous or Unsubstantiated Information

Meta‐researchers may encounter situations where interests appear relevant but cannot be independently substantiated [12, 13, 14]. For example, a publicly accessible payment database may list consulting fees paid to an author within a reporting period, while the corresponding publication contains no disclosure; although such a discrepancy warrants descriptive reporting, it does not by itself establish non‐disclosure or misconduct without confirmation that the payment met the journal's disclosure criteria at the time of submission.

Similarly, a meta‐researcher may observe that an author has led or authored a long‐standing body of work advocating a specific intervention or theoretical framework, while no non‐financial interests are disclosed in the publication. Such information may provide contextual insight and could signal a potential interest, but it should be reported descriptively, with explicit acknowledgement that professional commitment or intellectual investment alone does not constitute confirmed bias or a COI.

Existing reporting frameworks emphasise transparency but provide little guidance on how to handle such cases. In the absence of clear standards, cautious reporting is essential. Meta‐researchers should rely on verifiable sources, clearly distinguish confirmed disclosures from inferred or uncertain information, and avoid language that implies wrongdoing. Descriptive reporting preserves fairness while maintaining transparency, allowing readers to interpret patterns without being led toward unsubstantiated judgements.

## 6 Implications for Research Practice

Author identification in COI meta‐research should be understood as a methodological tool rather than a moral judgement [15]. When identification is necessary to support verification, contextual interpretation, or reproducibility, and when information is obtained from publicly accessible sources, naming authors may strengthen the credibility and usefulness of research.

When author identification is not central to the research question, anonymized or aggregate reporting may be sufficient and more proportionate. Journals and researchers should consider study aims, resource constraints and ethical implications when deciding whether to include individual‐level identifiers. Guidance at a field‐wide level would support consistent, ethically responsible practice, for example by recommending when verification of factual information is appropriate and how to report ambiguous interests.

Emphasising systemic improvement over individual blame aligns COI meta‐research with broader principles of research integrity. Understanding how disclosures are reported, how interests correlate with outcomes, and how transparency varies across disciplines allows the scientific community to design interventions that reduce bias and enhance accountability. Author identification, when used judiciously, can support these goals without assigning personal fault.

## 7 Conclusion and Key Message

Meta‐research on COI plays an important role in understanding how interests are disclosed and how they may influence scientific evidence. Decisions about author identification should be driven by methodological relevance and transparency needs, informed by ethical and practical considerations.

A cautious, proportionate approach to identification can enhance interpretability and verification while avoiding unnecessary reputational harm, supporting trust and accountability in research practice. Author identification in meta‐research on COI is a methodological tool, not a moral judgement. Developing clear, field‐wide guidance on when and how to identify authors—and how to report ambiguous or unsubstantiated information—can reinforce research accountability and improve trust in scientific evidence.

Transparent assessment and verification of COI not only supports methodological rigour but also directly informs the interpretation of clinical evidence. By ensuring that systematic reviews and guidelines are based on fully contextualised and verifiable data, COI transparency helps clinicians make better‐informed decisions, ultimately safeguarding patient care.

## Conflicts of Interest

The author declares no conflicts of interest.

## Data Availability

Data sharing not applicable to this article as no datasets were generated or analysed during the current study.
